# Supplementary material for: Molecular and Clinical Relevance of ZBTB38 Expression Levels in Prostate Cancer
Source: Cancers (Basel). 2020 Apr 29;12(5):1106. doi: 10.3390/cancers12051106 (PMC7281456; doi:10.3390/cancers12051106)
Supplement: Supplementary file 1 [file cancers-12-01106-s001.pdf]

Supplementary Materials: Molecular and clinical relevance of *ZBTB38* expression levels in prostate cancer.

Maud de Dieuleveult, Claire Marchal, Anne Jouinot, Anne Letessier and Benoit Miotto

Table S1. *ZBTB38* expression levels and *ETS* transcription factor gene fusions in localised prostate tumours.

Characteristics	Taylor et al. 2010 (GSE21032)				TCGA. 2015 (cBioPortal)				Ross-Adams et al. 2015 (GSE70770)			
	ZBTB38 expression (*N*)		Chi2	*p*-value	*ZBTB38* expression (*N*)		Chi2	*p*-value	*ZBTB38* expression (*N*)		Chi2	*p*-value
	Low	High			Low	High			Low	High		
**ERG fusion (by gene expression)**												
No fusion	34	30			-	-			29	26		
Fusion	30	35	0.626	0.428	-	-	-	-	24	28	0.462	0.496
**ERG fusion (by CGH*)**												
No fusion	45	33			-	-			-	-		
Fusion	13	17			-	-			-	-		
Flat	6	15	1.796	0.18	-	-	-	-	-	-	-	-
***ERG* fusion (by exon sequencing)**												
No fusion	-	-			57	72			-	-		
Fusion	-	-	-	-	88	72	3.34	0.067	-	-	-	-
***ETV1* fusion (by exon sequencing)**												
No fusion	-	-			141	140			-	-		
Fusion	-	-	-	-	4	5	0.236	0.626	-	-	-	-
**ETV4 fusion (by exon sequencing)**												
No fusion	-	-			135	138			-	-		
Fusion	-	-	-	-	10	7	0.114	0.734	-	-	-	-

N, number of patients per class. Significant *p*-value ($p < 0.05$) are highlighted in bold. * CGH: Comparative Genomic Hybridization.

Table S2. Correlation between *ZBTB38* expression levels and recurrent copy number aberrations (CNA) in localised prostate tumours.

Characteristics	TCGA. 2015 (cBioPortal)			
	ZBTB38 Expression (*N*)		Chi2	*p*-Value
	Low	High		
***BRCA1* CNA**				
Diploid	133	126		
Loss	12	19	1.769	0.183
***BRCA2* CNA**				
Diploid	116	126		
Loss	29	19	2.496	0.114
***CDK12* CNA**				
Diploid	137	138		
Loss	8	7	0.07	0.79
***CHD1* CNA**				
Diploid	113	132		
Loss	32	13	9.495	**0.002**
***FAM175A* CNA**				
Diploid	142	143		
Loss	3	2	0.203	0.651
***FANCC* CNA**				
Diploid	125	140		
Gain	16	4		

Loss	4	1	9.849	**0.007**
***FANCD2* CNA**				
Diploid	134	130		
Loss	11	15	0.067	0.410
***PTEN* CNA**				
Diploid	105	101		
Loss	40	44	0.268	0.604
***RAD51C* CNA**				
Diploid	136	141		
Loss	5	4		
Gain	4	0	4.201	0.122
***RB1* CNA**				
Diploid	90	118		
Loss	55	27	13.33	**0.0002**
***SPOPL* CNA**				
Diploid	115	138		
Loss	30	7	16.388	**0.00005**
***TP53* CNA**				
Diploid	98	100		
Loss	47	45	0.063	0.8

N, number of patients per class Significant p-value (p < 0.05) are highlighted in bold.

Table S3. Correlation between *ZBTB38* expression levels and recurrent mutations in localised prostate tumours.

**Characteristics**	**TCGA. 2015 (cBioPortal)**			
	ZBTB38* Expression *(N)		**Chi2**	***p*-Value**
	**Low**	**High**		
KMT2C				
No mutation	138	140		
Mutation	7	5	0.347	0.555
KMT2D				
No mutation	141	138		
Mutation	4	7	0.85	0.356
SPOP				
No mutation	121	138		
Mutation	24	7	10.438	**0.001**
TP53				
No mutation	132	138		
Mutation	13	7	1.933	0.164

Only recurrent mutations (i.e. present in > 10 samples) were evaluated.

N, number of patients per class. Significant *p*-value (*p* < 0.05) are highlighted in bold.

Table S4. Correlation between *ZBTB38* expression levels and molecular data in localised prostate tumours.

Characteristics	TCGA. 2015 (cBioPortal)			
	ZBTB38 Expression *(N)*		Chi2	*p*-value
	Low	High		
**RPPA clusters**				
Cluster 1	23	39		
Cluster 2	20	24		
Cluster 3	26	13		
Cluster 4	16	19		
Cluster 5	23	14	11.268	**0.023**
**DNA methylation cluster**				
Cluster 1	20	14		
Cluster 2#	65	31		
Cluster 3	38	50		
Cluster 4	22	49	25.001	**<0.00001**
**mRNA Cluster**				
Cluster 1#	64	37		
Cluster 2	59	38		
Cluster 3	22	70	36.80	**<0.00001**
**Integrative Cluster (iCluster)**				
Cluster 1	55	21		
Cluster 2	58	39		
Cluster 3	32	84	42.239	**<0.00001**

Unsupervised clustering of RPPA, DNA methylation and mRNA data were performed by the TCGA consortium (Ref. 6). #, SPOP mutations are associated with DNA methylation cluster 2 and mRNA cluster 1. *N*, number of patients per class. Significant *p*-value ($p < 0.05$) are highlighted in bold.

Table S5. Correlation between *ZBTB38* expression levels and chromosomal instability in metastatic prostate tumours.

**Characteristics**	**Robinson et al. 2015 (dbGap: phs000915.v1.p1)**				**Abida et al. 2019 (cBioPortal)**				**Kumar et al. 2016 (GSE77930)**			
	***ZBTB38* expression (*N*)**		**Chi2**	***p*-value**	***ZBTB38* expression (*N*)**		**Chi2**	***p*-value**	***ZBTB38* expression (*N*)**		**Chi2**	***p*-value**
	Low	*High*			*Low*	*High*			*Low*	*High*		
**Age (years)**												
<60	10	12			37	43			63	58		
>60	48	45	0.269	0.603	55	50	0.682	0.408	3	9	3.199	0.073
**Fraction genome altered**												
0 - 0.1	4	4			7	12			4	3		
0.1 - 0.5	39	34			64	69			35	45		
>0.5	16	21	1.018	0.601	35	24	3.549	0.169	28	19	3.116	0.21
**Mutation count**												
<50	7	9			34	31			37	40		
50 - 100	36	32			42	42			15	21		
>100	16	18	0.602	0.739	29	32	0.286	0.866	14	6	4.309	0.115

N, number of patients per class. Significant *p*-value ($p < 0.05$) are highlighted in bold.

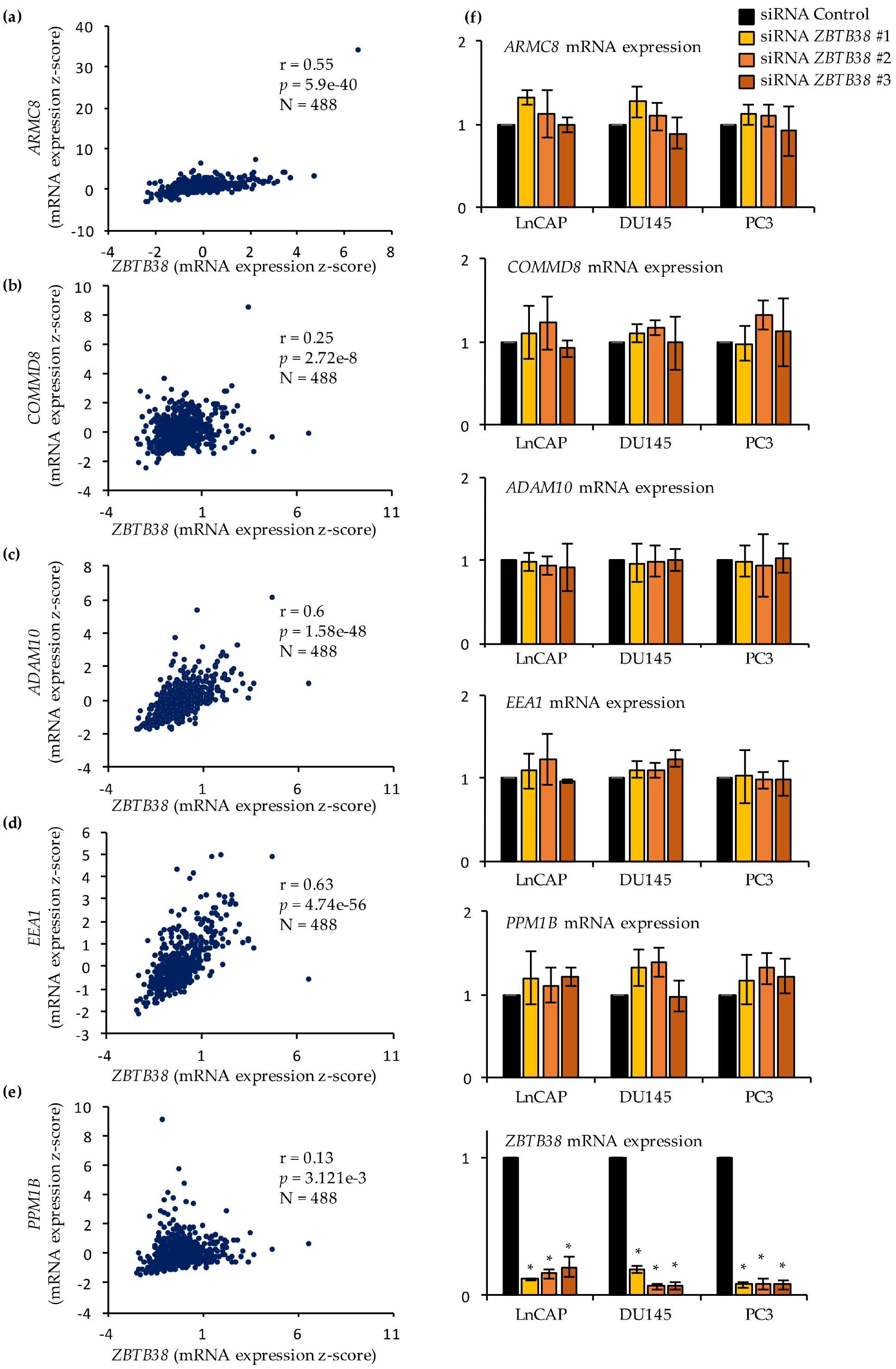

Figure S1. Correlation between *ZBTB38* expression and *ARMC8*, *COMMD8*, *ADAM10*, *EE1A* and *PPM1B* expression in prostate cancer; consequences of *ZBTB38* knock-down on gene expression. (**a**–**e**) Gene expression levels from TCGA (ref. 13) were plotted to illustrate the correlation between *ZBTB38* expression and: (**a**) *ARMC8*, (**b**) *COMMD8*, (**c**) *ADAM10*, (**d**) *EEA1* and (**e**) *PPM1B*. The r value indicates Pearson coefficients and the corresponding *p*-value are indicated. N, number of tumours analysed. (**f**) qRT-PCR analysis of *ARMC8*, *COMMD8*, *ADAM10*, *EEA1*, *PPM1B* and *ZBTB38* in LnCAP, DU145 and PC3 cells transfected with *ZBTB38* and control siRNAs ($n = 3$). *, $p < 0.05$ (vs. Control).

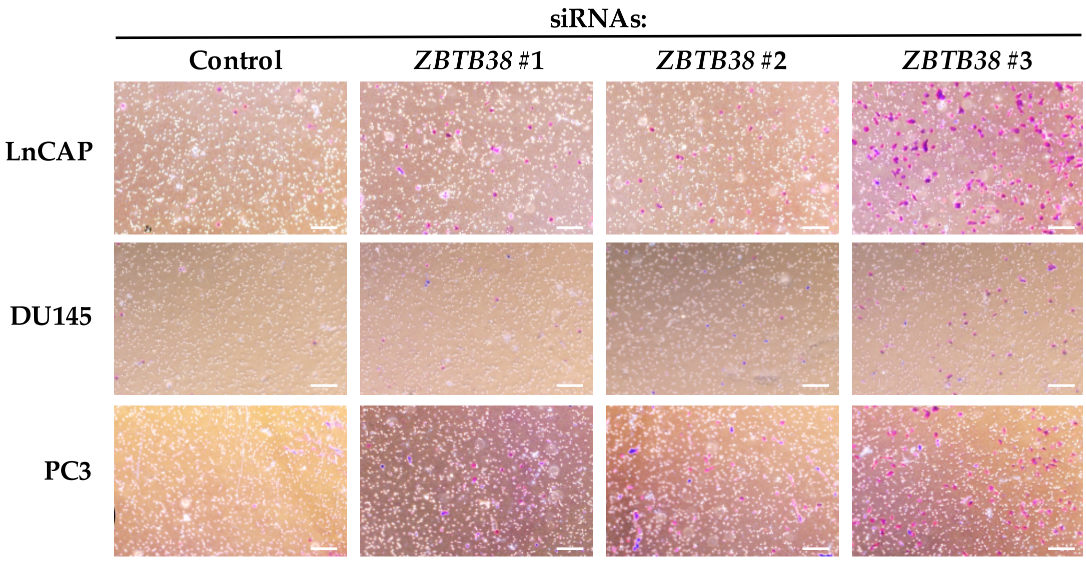

Figure S2. Representative images of transwell migration assays after transfection with control or *ZBTB38* siRNAs in LnCAP, DU145 and PC3 cancer cells. Cell numbers per field are presented in Figure 5c.

© 2020 by the authors. Submitted for possible open access publication under the terms and conditions of the Creative Commons Attribution (CC BY) license (http://creativecommons.org/licenses/by/4.0/).
